# Accessibility and factors associated with utilization of mental health services in youth health centers. A qualitative comparative analysis in northern Sweden

**DOI:** 10.1186/s13033-018-0249-4

**Published:** 2018-11-14

**Authors:** Isabel Goicolea, Cecilia Hultstrand Ahlin, Anna-Karin Waenerlund, Bruno Marchal, Monica Christianson, Maria Wiklund, Anna-Karin Hurtig, Miguel San Sebastian

**Affiliations:** 1 Unit of Epidemiology and Global Health, Department of Public Health and Clinical Medicine, Umeå University, Umeå, Sweden; 2 Department of Nursing, Umeå University, Umeå, Sweden; 3 Department of Public Health, Institute of Tropical Medicine, Antwerp, Belgium; 4 Unit of Physiotherapy, Department of Community Medicine and Rehabilitation, Umeå University, Umeå, Sweden

**Keywords:** Youth, Mental health, Youth health centers, Access, Qualitative comparative analysis, Sweden

## Abstract

**Background:**

Youth-friendly health care services can facilitate young people’s access to health care services and promote their health, including their mental health. In Sweden, a network of youth health centers exist since the 1970s, incorporated within the public health system. Even if such centers take a holistic approach to youth health, the focus has been in sexual and reproductive health care, and the extent of integrating mental health care services is less developed though it varies notably between different centers. This study aims to analyse the various conditions that are sufficient and/or necessary to make Swedish youth health centers accessible for mental and psychosocial health.

**Methods:**

Multiple case study design, using qualitative comparative analysis to assess the various conditions that makes a youth health center accessible for mental and psychosocial issues and mental health. The cases included 18 youth health centers (from a total of 22) in the four northern counties of Sweden.

**Results:**

In order to enhance accessibility for mental health services, youth health centers need to be trusted by young people. Trust was necessary but not sufficient, meaning that it had to be combined with other conditions: either having a team with a variety of professions represented in the youth health center, or being a youth health center that is both easy to contact and well-staffed with mental health professionals.

**Conclusions:**

Differentiated, first-line services for youth can play an important role in promoting youth mental health if certain conditions are fulfilled. Trust is necessary, but has to be combined with either multidisciplinary teams, or expertise on mental health and easy accessibility.

**Electronic supplementary material:**

The online version of this article (10.1186/s13033-018-0249-4) contains supplementary material, which is available to authorized users.

## Background

Despite youth being a time of strong potential, it is a period when individuals face substantial risk of morbidity and mortality associated with mental health problems [1]. Suicide is the third highest cause of death in people aged 10–19 years and depression the single largest contributor to the burden of disease for those aged 15–19 years [2]. Poor mental health is strongly connected with living conditions and psychosocial concerns, such as educational achievements, family relations, youth unemployment, alchol and drug abuse, and violence [3].

Mental health care services—ranging from community to specialized services—can promote youth mental health, detect mental health problems, and respond to youth’s needs in coordination with other sectors [3]. However, youths’ access to mental health care services is often hindered by factors such as self-reliance, concerns about confidentiality, and lack of trust [4–6]. Addressing such issues and ensuring that services comply with the World Health Organization (WHO) standards of being youth-friendly—namely accessible, available, equitable, acceptable and of good quality for diverse groups of youths [1]—might enhance youths’ effective utilization of health services, including for mental health [6]. From young people’s perspective, the following domains are central to ensuring youth-friendliness of services: accessibility of health care, staff attitude, communication, medical competence, age appropriate environments, and youth involvement [4]. Youth-friendly services should be based on supportive policies, assure confidentiality, coordinate with other services and develop outreach activities [7, 8]. In order for such services to be sustainable, the World Health Organization has recently highlighted the need to move forward from YFHS into youth responsive health systems [9]. However, despite the implementation of youth-friendly services is a cost-effective intervention that could contribute to better health among young people [10–13], in most countries health care for young people is usually an area not prioritized or where the focus is limited to sexual and reproductive health issues [1, 14–17]. The increase in mental health problems among young people in some (high income) countries, will hopefully result in putting youth-friendly services higher in the public agendas [3].

An important aspect in shaping utilization of services is how young people perceive the available health services [4, 18]. Since in many settings health-care services targeting young people have focused on somatic and/or sexual and reproductive health, they might not be perceived as places where mental health concerns are addressed [1, 6].

In Sweden, youth mental health is a concern [19, 20]. Mental problems have increased among mid-adolescents, especially girls [20]. Despite a decline among the general population, suicide rates remain stable among youth; with young men, LGBTQI + youth and persons born abroad are overrepresented [21]. In addition, health-related inequalities persist; girls and non-binary gender youths experience to a higher extent abusive treatment, discrimination and sexual abuse that might affect their mental health.

In Sweden, a network of around 300 specific youth-health services (called youth health centers—YHCs) has existed since the 1970s. YHCs aim to respond to the health-care needs of youths with a holistic approach to health. YHCs are generally located outside health-care facilities and staffed at a minimum with a midwife, counsellor and physician, although some YHCs also have other professionals, such as psychologists [22]. YHCs are part of the Swedish health system, publicly financed and managed. However, they are different from ordinary primary health care and specialized clinics, in that their focus is on youth. They work primarily with health promotion in all areas of youth health, the staff has a youth oriented perspective and the goal is to be able to help all young people with their questions. In case of specific somatic or psychiatric problems, youth can be referred to other services [23–25]. There is concordance between the Swedish YHC’s handbook published in 2015 and the WHO criteria for youth-friendliness, as described by Thomee et al. [25]. YHCs are well-known for the sexual and reproductive health services they offer, including the provision of contraceptives and testing for sexually transmitted infections. Currently, there is a national discussion as to whether YHCs should also provide first-line mental health care services [26].

Previous reports in Sweden have shown that YHCs are positively perceived by young people [27]. However, there are inequalities in terms of access for certain groups of youth [28] and for certain health issues: fewer youths perceive YHCs as places that address psychosocial issues in comparison to reproductive and sexual health issues. The number of staff and professions vary considerably between YHCs, i.e. in smaller settings is harder for YHCs to employ staff from diverse professional backgrounds [25]. Whether YHC’s characteristics influence how young people perceive and utilise them has not yet been studied.

## Aim

This study aims to analyse the various conditions that are sufficient and/or necessary to make Swedish youth health centers accessible for mental and psychosocial health.

## Methods

This study followed a multiple case study design to assess the various conditions that makes a YHC accessible for mental and psychosocial health. The cases included 18 YHCs (from a total of 22) located in 18 municipalities in the four northern counties of Sweden. Two YHCs refrained from participating and two did not provide enough data. These four counties encompass 44 municipalities, 60% of the country’s surface but only 12% of the population. The YHCs included varied in characteristics such as years of existence, targeted age groups and opening hours (see Additional file 1 for selected characteristics of the cases).

Information was collected from September 2016 to April 2017 via: (1) the YFHS-Swe questionnaire distributed by the YHC’s staff to young people visiting the YHC [29] (see Additional file 1 for more details and Table 1 for the characteristics of respondents) and (2) a document review complemented by email and telephone contacts with representatives of the YHCs.

**Table 1:** Selected characteristics of young people answering the questionnaire

	Percentage (total)
Type of visit	
Not first	83.6% (918)
First	14.4% (158)
Type of appointment	
Booked	72.9% (800)
Drop in	18.5% (203)
Went just there	5% (55)
Followed friend	1.8% (20)
Gender identity	
Woman	88.6 (973)
Man	8.5 (93)
Integrated	0.2 (2)
Non binary	0.5 (6)
Trans-experience	
Yes	1.4 (15)
No	95.4 (1047)
Sexual orientation	
Heterosexual	84.6 (929)
Homosexual	0.8 (9)
Bisexual	7.1 (78)
Queer	0.2 (2)
Asexual	0.1 (1)
Not sexual	2.1 (23)
Country of birth	
Sweden	93 (1021)
EU (not Sweden)	1.8 (20)
Other	4 (44)
Reason for consultation	
Contraceptives	44 (483)
Sexually transmitted infections: questions, testing	19.7 (216)
Physical problems	17.3 (190)
Psychological problems	14.8 (163)
Suspicion of pregnancy	9.8 (108)
Relations: friends, partners	2.7 (30)
Problems with family, parents	2.6 (29)
Questions on food, exercise, sleep	1.9 (21)
Questions related to sexual orientation, gender identity	1.7 (19)
Problems with work, studies	1.7 (19)
Drugs	0.4 (4)
Tobacco	0.2 (2)
Alcohol	0.1 (1)

### Qualitative comparative analysis using fuzzy sets

Qualitative comparative analysis (QCA) using fuzzy sets is a mixed methods approach developed by Ragin [30, 31]. It demands gaining familiarity with each case (qualitative approach), and allows to simplify huge amounts of data looking for patterns or causal pathways (quantitative approach), and finally interpret these patterns going back to the data generated from each case [31]. As Ragin puts it, the underlying idea is to identify a ‘causal recipe’, a specific combination of causally relevant ingredients linked to an outcome [30, 31]. Two core tenets of QCA’s approach to causation are that (1) outcomes are produced by conditions that do not work in isolation but complement each other (conjunctural causation), and (2) there may be more than one causal combination that explains an outcome (equifinal causation). From each case information in regards to outcomes and conditions (factors that in combination can cause the outcome) is gathered, and potential combination of conditions and outcomes are then assessed in each case. Through Boolean algebra potential the combinations of conditions that are most frequently present when the outcome is present are identified. Finally, such combinations are interpreted going back to the data [30, 31].

QCA uses Boolean algebra to assess to what extent a configuration of conditions explains outcomes in terms of necessity—whether the cause is present in all (or almost all) the instances of the outcome—and sufficiency, i.e. whether the cause is invariably (or almost) followed by the outcome. Through the notions of ‘necessity’ and ‘sufficiency’, the researcher assesses the consistency of conditions or combinations of conditions: the higher the consistency of a set of conditions, the better it explains the outcome [31, 32]. In terms of practical implications, the criterion of ‘coverage’ is important: to what extent is the outcome covered by the conditions- or what is the proportion of cases exhibiting the combinations that are being assessed [31, 32]?

QCA can be used with dichotomous variables (crispy sets) but also with numeric values (fuzzy sets). With fuzzy sets, the data need to be calibrated first by establishing three cut-off points to decide whether a case is totally Yes, partially Yes or totally No for a specific condition (called ‘the degree of membership’ in QCA terms) [31, 32].

### Identifying and assessing the outcome of interest

We defined ‘access to mental health’ as the outcome of this study. The data source for the outcome was the YFHS-Swe questionnaire, answered by 1098 young people coming to the YHCs. This questionnaire gathers information about 13 domains of youth friendliness, including ‘access to mental health’, and is based on a validated version of the YFHS-WHO + questionnaire [29]. The domain ‘access to mental health’ encompasses 12 items, assessed via a Likert-type scale (0–5), which explore to what extent a young person considers that he/she can get help for certain types of mental health issues in that specific YHC (see Additional file 2 for a list of the items included). The items refer not only to mental health problems, but also to adverse events or other psychosocial aspects that could pose a risk to mental health and well-being. Aggregated scores and means were calculated for each of the YHCs.

### Identifying and assessing the conditions

To identify relevant conditions that, from the perspective of youth, might make a YHC accessible for mental health issues, an initial literature and document review was conducted. This was not a systematic review of the literature and documents. However, we searched the literature and existing reports and documents in order to identify elements that have been found as relevant for enhancing youth-friendliness in general and for aspects related with mental health in particular. Documents that were studied included published scientific articles from the field of youth friendliness, guidelines from the World Health Organization [7] and the handbook from the Swedish organization of YHCs [24]. Twenty-three preliminary conditions were first selected, and afterwards reduced to ten conditions, by eliminating those that were not feasible to assess, and by collating some that were closely related, e.g. “separate YHC from other healthcare services” and “good location” since both addressed the issue regarding where the YHC is located. This process was conducted by three of the authors of the manuscript: CHA, AKW and IG.

The ten conditions that were considered to be most relevant were: (1) non-judgement; (2) respect; (3) privacy; (4) trust; (5) easiness to contact; (6) collaboration with schools; (7) outreach activities; (8) long opening hours; (9) multidisciplinary team; and (10) expertise on mental health.

In QCA, it is recommended not to test a large number of conditions in the actual analysis, especially when the number of cases is limited, in order to avoid having too many combinations of conditions that are not present in any of the cases [30–32]. To this end, we reduced the ten conditions to four. The criteria for dropping conditions were: (1) when there were several conditions assessing similar aspects, the one that we considered more comprehensive was kept, i.e. we drop “long opening hours” and kept “easiness to make contact”, (2) we dropped “collaboration with schools” and “outreach activities” because we were not convinced that the information gathered allowed us to assess diverse degrees in the cases, and (3) we excluded ‘non-judgement’, ‘respect’ and ‘privacy’, since all YHCs scored very high for these conditions with almost no variation between YHCs.

The conditions we thus included in each case were:

1. Trust—whether young people perceived that they could trust the YHC staff.
2. Multidisciplinary team—whether there is a wide range of professionals at the YHC.
3. Expertise in mental health—whether at least one of the staff has specific expertise in mental health.
4. Easiness to make contact—whether young people perceived that they could contact the YHC easily.

We refer to Table 2 for a summary of how the conditions and the outcome were operationalized and assessed, and Additional file 2 for a more detailed description of the items used to assess certain conditions and the outcome.

### Data analysis

After the data were gathered for the conditions and outcome from each YHC, calibration was conducted for the numeric values (the outcome, and the conditions of trust and easiness to contact) in order to be able to conduct the fuzzy set QCA [30–32]. We performed the calibration with the help of the software program fzQCA, assigning the highest, middle and lowest values. Theoretical assumptions and knowledge of the cases were used to establish the three cut-off points. A raw table with calibrated values can be found in Table 3.

**Table 2:** Conditions and outcome, descriptors and data collection techniques

Sets (conditions/outcome)	Descriptors and source^a^	Possible answers and value for each answer	Abbreviation
Condition: Trust	Do the young people perceive that they can trust the YHC, staff and consultation? YFHS-Swe questionnaire (3 items)	Mean value from the three items Numeric value between 1 (lowest) and 5 (highest)	Trust
Condition: Multidisciplinary team	Does the YHC team consist of a variety of professions? Documentary review, contact YHCs	Number of different professions represented in the team of the YHC (medical doctor, nurse, midwife, social worker, psychologist, nutritionist, gynaecologist) Numeric value from 2 to 8	Multiprof
Condition: Expertise in mental health	Does the staff have special competence in mental health? Documentary review, contact YHC	0 or 1 0 YHC has curator with no further training on mental health, no psychologist, no psychiatric nurse or no curator 1 YHC has psychologist, psychiatric nurse or curator with extra training on mental health	Mentprof
Condition: Easy to contact	Is the YHC and its staff perceived as easy to get in contact with? YFHS-Swe questionnaire (5 items)	Mean value from the five items Numeric value between 1 (lowest) and 5 (highest)	Contact
Outcome: A YHC that youth perceive they can come for mental health issues	Is the YHC perceived as a place to come for mental health issues YFHS-Swe questionnaire (12 items)	Mean value from the 12 items Numeric value between 1 (lowest) and 5 (highest)	Mentaccess

^a^For a more detailed description of the items included in outcomes and conditions see Table 1

First, the raw table was imported into the software fsQCA in order to analyse necessary conditions, namely conditions that are present in all the instances of the outcome, and thus in all the combinations. We calculated consistency and coverage for the four selected conditions, using a cut-off point of 0.9 and 0.8, respectively, for labelling a condition as necessary, as suggested in the literature [32]. Conditions identified as necessary were plotted on an XY diagram and excluded from the truth table, since they would appear in all the combinations.

Second, we proceeded to develop a truth table. The truth table displays all the possible combinations of conditions, the number of cases where each combination is present (coverage), and the degree of consistency of a positive outcome for each combination. From this truth table, we eliminated the inconsistencies—i.e. configurations of conditions with less than one case—and we reset the outcome to 1 if consistency was higher than 0.8. Subsequently, a standard analysis was applied, and the intermediate solution formula was chosen [30, 31]. The solution formula presents the combinations that produce the outcome in a consistent way. Usually, more than one combination of conditions emerge, and for each of them, consistency and coverage scores are calculated. If a combination of conditions has a consistency of 1, this means that such a combination always leads to the outcome. If a combination of conditions has a coverage of 1, this means that this combination is able to explain all of the occurrences of the outcome.

## Results

We present the truth table in Additional file 3 the assessment of necessity in Table 4, and the intermediate solution formula and parameters in Table 4.

**Table 3:** Raw table with calibrated values for conditions and outcome

Case	Trust	Multiprof	Mentprof	Contact	Mentaccess^a^
1	0.96	1	1	0.7	0.88
2	0.95	0.96	0	0.8	0.9
3	0.99	0.57	0	0.01	0.97
4	0.99	0.96	1	0.98	0.96
5	0.66	0.18	0	0.07	0.36
6	0.92	0.57	1	0.81	0.59
7	0.94	0.96	1	0.61	0.87
8	0.94	0.18	0	0.32	0.96
9	0.91	0.57	0	0.33	0.86
10	0.95	0.18	0	0.17	0.44
11	1	0.96	1	0.72	0.83
12	0.95	0.96	1	0.68	0.9
13	0.98	0.18	1	0.89	0.93
14	0.99	0.96	1	0.89	0.95
15	0.94	0.57	1	0.74	0.9
16	0.93	0.57	0	0.3	0.88
17	0.97	0.96	1	0.64	0.92
18	0.62	0.18	0	0.05	0.44

^a^Values for each condition and outcome range from 0 (no membership) to 1 (full membership into the set)

**Table 4:** Analysis of potential necessary conditions for Mentaccess

	Consistency^a^	Coverage^a^
Trust	1	0.88
Multiprof (multi professional team)	0.73	0.92
Mentprof (professional with expertise in mental health)	0.55	0.86
Contact (easy to contact the YHC)	0.67	0.98

^a^Values range from 0 to 1. A consistency of 1 means that in all the cases that fulfil this condition the outcome is present. A coverage of 1 means that in all cases were the outcome is present, the condition is exhibited

The analysis of necessary conditions showed that trust had a consistency of 1 and a coverage of 0.88, meaning that it was a necessary condition and thus it was excluded from the calculations of the solution formula (Table 4), as it will appear in all the possible solutions.

However, trust was not enough for achieving good access for mental health, and other conditions were needed in order to ensure that young people perceived that YHCs were accessible.

The XY plot between trust and the outcome ‘Mentaccess’ showed that almost all cases were located in the lower triangle, a pattern consistent with necessity (the outcome is a subset of the condition) (Additional file 4).

Two combinations of conditions showed good consistency and acceptable coverage: (1) having a team with a large number of different professions represented (‘Recipe’ 1 with a consistency of 0.96 and raw coverage of 0.75), and (2) being perceived as easy to contact and having professional(s) with expertise of mental health within the team (‘Recipe’ 2 with consistency of 0.97 and coverage of 0.52) (Table 5).

The solution formula (both recipes combined) had a consistency of 0.93, meaning that it produced the outcome most of the times. The coverage was 0.78, indicating that 14 out of the 18 cases were covered by the solution formula.

Table 4 shows the two main combinations of conditions that, together with trust, made a YHC more accessible for mental health issues from the perspective of young people. Having a larger variety of professions working at the same YHC explained on its own why four YHCs were doing well in ‘Mentaccess’. Easiness to contact and having at least one professional with specific expertise in mental health issues explained good ‘Mentaccess’ on its own in one case. For nine cases, the explanation for being perceived as having good accessibility was a combination of having diverse professions represented, being perceived as being easy to get in contact with and having at least one professional with specific expertise in mental health, in addition to being perceived as a YHC that young people could trust.

Only one case remained unexplained by this solution formula: case number 8. This YHC was perceived as accessible for mental health issues, despite not having a team with a large number of professions represented, and not combining with being perceived as easy to contact and having at least one professional with specific expertise in mental health. However, the necessary condition of trust was present in this YHC, meaning that trust interacted with other conditions not captured by this study to make this specific YHC still accessible for mental health issues.

**Table 5:** Parameters of the two pathways and solution formula

	“Recipe” 1	“Recipe” 2
	Multiprof	Contact * Mentprof
Consistency	0.96	0.97
Raw coverage (# of cases)	0.75 (13)	0.52 (10)
Unique coverage (# of cases)	0.51 (4)	0.06 (1)
ID of cases explained	1, 2, 3, 4, 6, 7, 9, 11, 12, 14, 15, 16, 17	4, 6, 7, 11, 12, 13, 14, 15, 17
ID of cases explained uniquely	2, 3, 9, 16	13
# unexplained cases	Two (cases 8 and 13)	Five (cases 2, 3, 8, 9, 16)

**Fig. 1:**
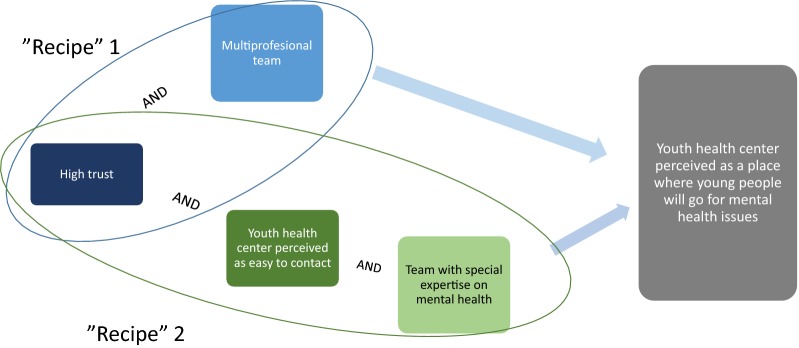
Summary of combination of conditions (“recipes”) for mental health accessibility of Swedish youth health centers

Figure 1 summarizes the two ‘recipes’ that make a YHC accessible for mental health issues. Note the key role of the necessary, but not sufficient, trust condition.

## Discussion

This study shows that in order to enhance the accessibility concerning mental health issues, YHCs need to be trusted by young people. Trust was necessary but not sufficient, meaning that it had to be combined with other conditions: either having a team with a variety of professions represented in the YHC, or being a YHC that is both easy to contact and has one (or more) professional(s) with specific expertise in mental health.

Interpersonal trust in health-care professionals and institutional trust in the health system enable relationships that produce positive health outcomes, and contribute to generating wider social value [33]. The literature highlights that on the one hand, young people have lower trust in health care [4, 34–37] and that on the other hand, trust is especially relevant for ensuring that young people access health-care services in general [36, 37] including for mental health issues [6]. This study underlines the crucial role of trust, and makes two important points: (1) the YHCs studied were, in general, trusted by young people (16 out of 18), and (2) strengthening youths’ trust in YHCs will enhance their accessibility, not only for issues that are considered as the ‘traditional’ domains of YHCs (namely sexual and reproductive health), but also for mental health.

Our results might reflect that youth appreciate the care provided and this contributes to building trust, so that they return to the YHC. The literature provides evidence of the crucial role of a positive first encounter and continuity of care in building trust with young people, and points out that differentiated services (such as YHCs) might facilitate the establishment of trust between providers and young users [5, 18, 34, 35]. Findings from this study also point out a critical challenge: how to ensure that a more diverse group of youths (for example, immigrant youths, LBGTQI + youths) trusts YHCs enough to get in contact and receive care that strengthens their trust and makes them return. This becomes especially relevant if we consider that unaccompanied refugees, asylum-seekers and LBGTQI + youths have been shown to be at higher risk of experiencing mental health problems and are perceived as accessing YHCs less often [38, 39].

Trust alone is not enough: case 10 shows that high trust in the absence of the other conditions leads to low accessibility for mental health. While building trust is very much dependent on the micro-level encounters between youth and the health professionals [5, 18, 36, 37], the other three conditions (having multi-professional teams, being easy to contact and having expertise on mental health) depend much more on the organization of the YHCs and the resources they receive. Other studies in Sweden have shown that YHCs look different depending on their geographical location, i.e. big urban areas versus smaller villages, different counties. This flexibility allows them to adapt services to local realities but brings the risk of inequalities [25]. A recent mapping highlights the huge differences between YHCs when it comes to specific resources for mental health.

The conditions assessed are certainly easier to ensure in YHCs with more resources: all the YHCs that displayed both combinations of conditions were open more than 20 h/week, and only three of the nine were located in rural municipalities. However, among YHCs displaying only one of the recipes, there were more smaller YHCs (four out of the five were open less than 10 h/week), and only one of them was located in a middle-sized town. This demonstrates that even in smaller municipalities with fewer resources, there are strategies that can be implemented in order to enhance accessibility for mental health. This contrasts with findings from the few studies on access to (mental) health-care services for rural youth, which highlight the challenges for ensuring sustainable services for youths in rural and smaller municipalities [18, 40].

Two points are especially relevant here and deserve further research. The first relates to the three YHCs (cases 4, 11 and 14) that, despite being located in less populated rural municipalities, were able to ensure a multi-professional team, being easy to contact (probably through being open more than 20 h/week), and having a professional with expertise in mental health. How they have managed to do this, and how and why they might have attracted more resources from the regional government, deserves further investigation.

The second point relates to the only exception in our data set: case number 8 is a small YHC with a team of two (so, not fulfilling the Swedish Association of Youth Centres criteria for being a YHC), no specific expertise in mental health, and not easy to contact (it opens only 4 h/week), but with high trust and high accessibility for mental health. A possible explanation might be that the midwife working in this YHC also addressed mental health issues—in fact, in 21% of the consultations with the midwife, psychosocial issues were discussed, which aligns with the holistic approach, where youth and not a specific health problem is put at the centre of attention, and where midwives have been known to play a key role beyond sexual and reproductive health issues [24, 25, 27]. There is not only a need for posting professionals with specific expertise in mental health within all YHCs, but also to build upon the holistic approach in which every professional at the YHCs addresses youth health, including mental health.

Beyond the Swedish context, this study highlights that implementing a network of health care services that are youth-centred (such as Swedish YHCs) makes the implementation of mental health care services for young people easier than if such services have to be implemented without such a basic ground. In addition, it points out that if mental health care services aim to attract young people, they have to plan for strategies to enhance youths’ trust towards such services. The strategies that have been used by Swedish YHCs to enhance young people’s trust, can be useful to other countries that are currently engaged in implementing first line mental health care services for young people [41]. The study also underlines that making health care services accessible for mental health issues demands political commitment in terms of a sustained provision of resources, and in terms of a flexible approach that allows for different models of service provision to coexist, depending on contextual circumstances, i.e. rural settings vs big cities. Such recommendations are especially relevant for other high-income countries with a considerable proportion of rural population, such as other Scandinavian countries, Australia, or Canada.

### Limitations

The number of cases in the study is a limitation; with more cases, it is more likely that we could have found larger variation in outcomes and in certain conditions. We had to drop some conditions that might have been relevant for enhancing accessibility for mental health (i.e. privacy, non-judgemental attitudes), because all the YHCs scored highly. That does not mean that these conditions are not relevant, but merely that we could not explore them with the sample of cases that we had. However, we also have to state that we were able to include the majority of YHCs existing in this region (18 out of 22). A major limitation is that we are evaluating YHCs from the perspective of those coming to the YHCs, thus certain subgroups of youth are underrepresented [25, 28]. In addition, the fact that the staff of the YHC was responsible for offering the questionnaire to the young people might have also affected who was approached and their answers (through social desirability). Finally, the study is conducted in a very unique context—a high income country with a well-developed health system, where youth-friendly services have been working for more than 40 years and are an undisputable part of the system. The transferability of these findings to low-middle income settings or countries with a less developed public health care system is arguable. Still, some aspects might be relevant, despite poor resources, such as the importance of building trust, offering easy access and an holistic approach with competence in mental health.

## Conclusion

In order to ensure that differentiated health care services for young people are accessible for mental health issues they need to be trusted by young people and either (1) ensure a multidisciplinary team or (2) ensure that they are easy to get in contact with, and they are well-staffed with mental health professionals.

The resources needed to ensure that YHCs have multidisciplinary teams, at least one professional with expertise in mental health, and are easy to contact by youths, do not demand a large investment. This study shows that even in rural and sparsely populated municipalities, characteristic of northern Sweden, but also of other parts of the EU, USA, Australia and Canada, services that are accessible for youth mental health can be organised.

## Additional files

**Additional file 1.** Selected characteristics of cases.

**Additional file 2.** Items from the YFHS-Swe questionnaires used for conditions (easy to contact and trust) and outcome (mental access).

**Additional file 3.** Truth table.

**Additional file 4.** XY plot showing relationship consistent with necessity.
