# Supplementary material for: Accessibility and factors associated with utilization of mental health services in youth health centers. A qualitative comparative analysis in northern Sweden
Source: Int J Ment Health Syst. 2018 Nov 14;12:69. doi: 10.1186/s13033-018-0249-4 (PMC6234690; doi:10.1186/s13033-018-0249-4)
Supplement: Supplementary file 1 — Additional file 1. Selected characteristics of cases. [file 13033_2018_249_MOESM1_ESM.docx]

| **Additional file 1. Selected characteristics of cases** | | | | | | | |
| --- | --- | --- | --- | --- | --- | --- | --- |
| **Case** | **Region** | **Type of municipality** | **YC open since** | **Opening hours** | **Numbers of days per week open** | **Type of professions represented at YC** | **Number of questionnaires collected** |
| 1 | A | B3. Medium-sized town | 1993 | 45h/week | 5 | midwife, physician, psychologist, curator, psychotherapist, psychiatric nurse, district nurse, dietitian, social worker, receptionist, | 82 |
| 2 | A | C6. Small towns - municipalities with a population of at least 15 000 inhabitants in the largest urban area | 1984 | 40 h/week | 5 | midwife, physician, curator | 96 |
| 3 | A | C8. Rural municipalities | 2001 | 4h 30 min/week | 1 | midwife, physician, curator, social worker | 26 |
| 4 | B | C8. Rural municipalities | 1975 | 30h 30min/week | 5 | midwife, physician, assistant nurse | 100 |
| 5 | B | C8. Rural municipalities | 1992 | 2h 45 min/week | 1 | curator, midwife | 45 |
| 6 | B | C6. Small towns - municipalities with a population of at least 15 000 inhabitants in the largest urban area | 1992 | 34h/week | 5 | midwife, physician, curator | 81 |
| 7 | B | C6. Small towns - municipalities with a population of at least 15 000 inhabitants in the largest urban area | 1993 | 33h/week | 5 | midwife, physician, curator | 86 |
| 8 | B | B5. Commuting municipalities with a low commuting rate near medium-sized towns | 2001 | 4h 30min/week | 1 | midwife, curator | 24 |
| 9 | C | C6. Small towns - municipalities with a population of at least 15 000 inhabitants in the largest urban area | NA | 11h/week | 3 | midwife, physician, curator | 81 |
| 10 | C | C8. Rural municipalities | NA | 6h/week | 1 | midwife, curator | 12 |
| 11 | C | C8. Rural municipalities | 1989 | 21h/week | 3 | midwife, physician, curator, assistant nurse | 41 |
| 12 | C | B3. Medium-sized town | 1976 | 35h/week | 5 | midwife, physician, curator | 95 |
| 13 | C | B4. Commuting municipalities near medium-sized towns | NA | 6h 15min/week | 1 | midwife, curator | 19 |
| 14 | C | C8. Rural municipalities | 1984 | 21h/week | 3 | midwife, physician, curator, assistant nurse | 49 |
| 15 | C | C6. Small towns - municipalities with a population of at least 15 000 inhabitants in the largest urban area | 1994 | 35h 30min/week | 5 | midwife, physician, curator | 98 |
| 16 | D | C9. Rural municipalities with a visitor industry | 1992 | 8h/week | 1 | midwife, physician, curator | 35 |
| 17 | D | B3. Medium-sized town | 1989 | 36h 30min/week | 5 | midwife, physician, curator, assistant nurse | 116 |
| 18 | B | B5. Commuting municipalities with a low commuting rate near medium-sized towns | 2007 | 10h/week | 4 | midwife, physician, curator, school nurse | 12 |
