# Supplementary material for: Accessibility and factors associated with utilization of mental health services in youth health centers. A qualitative comparative analysis in northern Sweden
Source: Int J Ment Health Syst. 2018 Nov 14;12:69. doi: 10.1186/s13033-018-0249-4 (PMC6234690; doi:10.1186/s13033-018-0249-4)
Supplement: Supplementary file 2 — Additional file 2. Items from the YFHS-Swe questionnaires used for conditions (easy to contact and trust) and outcome (mental access). [file 13033_2018_249_MOESM2_ESM.docx]

Additional file 2. Items from the YFHS-Swe questionnaires used for conditions (easy to contact and trust) and outcome (mental access).

| Question | Abbreviation |
| --- | --- |
| What do you think of the opening hours of this service? | Contact |
| What do you think about the possibility of getting a booked appointment here? | Contact |
| What do you think about the time you had to wait in the waiting room? | Contact |
| What do you think about the possibility to be in contact, by phone, with this service? | Contact |
| What do you think about the possibility to be in contact with the staff here (personally or by phone)? | Contact |
| Mental problems, for example, worry, feeling blue, stress, depression, anxiety, thoughts about hurting yourself or suicidal thoughts | Mentaccess |
| Questions concerning questions related to sexual orientation or gender identity | Mentaccess |
| Concerning relationship to friend/partner | Mentaccess |
| Smoking or help to stop smoking cigarettes | Mentaccess |
| Problems with alcohol | Mentaccess |
| Problems with marijuana or other drugs | Mentaccess |
| Problems with parents or family | Mentaccess |
| Problems with work/unemployment, school or university | Mentaccess |
| Questions about food, exercise or sleeping habits | Mentaccess |
| Concerning sexual actions that have occurred against your will | Mentaccess |
| Concerning that someone has, or tried to, hurt you, or that you did it to someone else, for example fights, hits or kicks | Mentaccess |
| Concerning that someone made you feel bad with something they said, for example threatened or insulted you | Mentaccess |
| In regards to todays’ visit. How much trust do you have in this person? | Trust |
| How much do you trust that the ones working in this service will not talk about your problems with other adults, for example your parents, if you don´t agree to it? | Trust |
| How much do you trust that the staff will keep your problems confidential, i.e. so that no one else gets to know about your problems? | Trust |
