# Supplementary material for: Accessibility and factors associated with utilization of mental health services in youth health centers. A qualitative comparative analysis in northern Sweden
Source: Int J Ment Health Syst. 2018 Nov 14;12:69. doi: 10.1186/s13033-018-0249-4 (PMC6234690; doi:10.1186/s13033-018-0249-4)
Supplement: Supplementary file 3 — Additional file 3. Truth table. [file 13033_2018_249_MOESM3_ESM.docx]

| Multiprof | Mentprof | Contact | Number of cases | Mentaccess | Raw consistency |
| --- | --- | --- | --- | --- | --- |
| 1 | 0 | 1 | 1 | 1 | 1 |
| 0 | 1 | 1 | 1 | 1 | 1 |
| 1 | 0 | 0 | 3 | 1 | 1 |
| 1 | 1 | 1 | 9 | 1 | 1 |
| 0 | 0 | 0 | 4 | 0 | 0.72 |

Additional file 3. Truth table
