# Supplementary material for: Accessibility and factors associated with utilization of mental health services in youth health centers. A qualitative comparative analysis in northern Sweden
Source: Int J Ment Health Syst. 2018 Nov 14;12:69. doi: 10.1186/s13033-018-0249-4 (PMC6234690; doi:10.1186/s13033-018-0249-4)
Supplement: Supplementary file 4 — Additional file 4. XY plot showing relationship consistent with necessity. [file 13033_2018_249_MOESM4_ESM.docx]

Additional file 4. XY plot showing relationship consistent with necessity

mentaccess

trust
